# Effects of dietary patterns and low protein intake on sarcopenia risk in the very old: The Newcastle 85+ study

**DOI:** 10.1016/j.clnu.2019.01.009

**Published:** 2020-01

**Authors:** Antoneta Granic, Nuno Mendonça, Avan A. Sayer, Tom R. Hill, Karen Davies, Mario Siervo, John C. Mathers, Carol Jagger

**Affiliations:** a AGE Research Group, Institute of Neuroscience, Newcastle University, Newcastle Upon Tyne, United Kingdom; b NIHR Newcastle Biomedical Research Centre, Newcastle Upon Tyne Hospitals NHS Foundation Trust and Newcastle University, Newcastle Upon Tyne, United Kingdom; c Newcastle University Institute for Ageing, Newcastle Upon Tyne, United Kingdom; d Institute of Health & Society, Newcastle University, Newcastle Upon Tyne, United Kingdom; e Human Nutrition Research Centre, Newcastle University, Newcastle Upon Tyne, United Kingdom; f Institute of Cellular Medicine, Newcastle University, Newcastle Upon Tyne, United Kingdom

**Keywords:** Aged 80 and over, Dietary patterns, Low protein intake, Newcastle 85+ study, Sarcopenia, aBW, adjusted body weight, DPs, dietary patterns, EWGSOP, European Working Group on Sarcopenia in Older Adults, GS, grip strength, SMI, skeletal muscle index, TUG, Timed Up-and-Go test

## Abstract

**Background:**

Sarcopenia, a progressive age-related loss of skeletal muscle mass and strength, leads to disability, falls, and hospitalisation. Individual variation in sarcopenia onset may be partly explained by lifestyle factors such as physical activity and diet. Healthy dietary patterns (DPs) have been linked to better physical functioning in older adults, but their role in sarcopenia in the very old (aged ≥85) is unknown.

**Aims:**

To investigate the association between DPs and the risk of sarcopenia over 3 years, and to determine whether protein intake influences this relationship in community-dwelling older adults from the Newcastle 85 + Study.

**Methods:**

The analytic sample consisted of 757 participants (61.2% women) who had dietary assessment at baseline. After two-step clustering with 30 food groups to derive DPs, we used logistic regression to determine the risk of prevalent and incident sarcopenia across DPs in all participants, and in those with low (<1 g/kg adjusted body weight/day [g/kg aBW/d]) and good protein intake (≥1 g/kg aBW/d).

**Results:**

We identified three DPs (DP1: ‘Low Red Meat’, DP2: ‘Traditional British’ and DP3: ‘Low Butter’) that varied by unsaturated fat spreads/oils, butter, red meat, gravy and potato consumption. Compared with participants in DP3, those in DP2 had an increased risk of prevalent (OR = 2.42, 95% CI: 1.15–5.09, p = 0.02) but not 3-year incident sarcopenia (OR = 1.67, 0.59–4.67, p = 0.33) adjusted for socio-demographic, anthropometry, health and lifestyle factors. Furthermore, DP2 was associated with an increased risk of prevalent sarcopenia at baseline (OR = 2.14, 1.01–4.53, p = 0.05) and 3-year follow-up (OR = 5.45, 1.81–16.39, p = 0.003) after adjustment for key covariates in participants with good protein intake.

**Conclusion:**

A DP high in foods characteristic of a traditional British diet (butter, red meat, gravy and potato) was associated with an increased risk of sarcopenia even when overall protein intake was good. The results need to be replicated in other cohorts of the very old to understand the role of DPs in sarcopenia onset and management.

## 1 Introduction

The European Working Group on Sarcopenia in Older Adults (EWGSOP) defines sarcopenia as a progressive and generalised age-related loss of muscle mass and strength [1], which starts in the fifth decade of life and increases in prevalence to ∼12.5–50.0% in older adults aged >80 [2], [3], [4], [5]. Clinically, sarcopenia is linked with numerous adverse health events including osteoporosis, diabetes, and obesity [6], [7], [8], [9], and strongly associated with frailty, risk of falls, and mortality in mid and late adulthood [10], [11], [12], [13]. Whilst declines in muscle mass and strength are a common features of ageing [14], the rate of the loss varies substantially between individuals, and has been attributed to intrinsic (e.g. hormonal changes, genetic factors) [15] and extrinsic (environmental) factors such as physical activity and diet [16], [17], [18].

Higher physical activity and resistance exercise are established modifiable lifestyle factors that minimise muscle mass/strength decline [20], [21], alone or in combination with higher protein intake (≥1 g/kg body weight (BW)/day) [16], [22], [23]. In comparison, the role of the whole diet in sarcopenia risk has been little researched, with some evidence linking higher intake of individual nutrients (e.g. protein, vitamin D, n-3 polyunsaturated fatty acids (n-3 PUFA), antioxidants) [24], [25], [26], [27], [28] and food groups (e.g. meats, fruits and vegetables, dairy) [29], [30] to reduced risk of sarcopenia and better muscle function in older adults. However, only a few studies have been conducted in the very old (aged ≥85) [31], [32], who have higher prevalence of sarcopenia [4], and are at increased risk of malnutrition [33], poor diet [34], [35], [36] and inactivity [37]—the main modifiable risk factors for loss of muscle mass/strength.

The derivation of dietary patterns (DPs) has been successfully used to characterise diet complexity and quality, accounting for the synergy between foods and nutrients, and to examine the association of DPs with various health outcomes [38], [39]. Two different approaches for assessing DPs have been used widely: (a) dietary scores or indices which are based on the prevailing hypotheses of what constitutes a healthy diet for disease risk reduction (e.g. Mediterranean diet score), and (b) factors or clusters derived using data reduction methods on available dietary data without prior hypotheses about diet-disease relationship. A few studies have utilised these methods to investigate associations between DPs and sarcopenia (e.g. [40], [41], [42], reviewed in [43], [44])— the Mediterranean DP has been the most common approach and none of the studies has been conducted in the very old.

Therefore, we aimed to: (a) derive and characterise DPs in community-dwelling very old adults from the Newcastle 85 + Study at baseline; (b) determine the risk of prevalent and incident sarcopenia over 3 years in relation to DPs, and (c) explore whether the DP-sarcopenia relationship is influenced by participants’ protein intake.

## 2 Materials and methods

### 2.1 Study design and participants

**Fig. 1:**
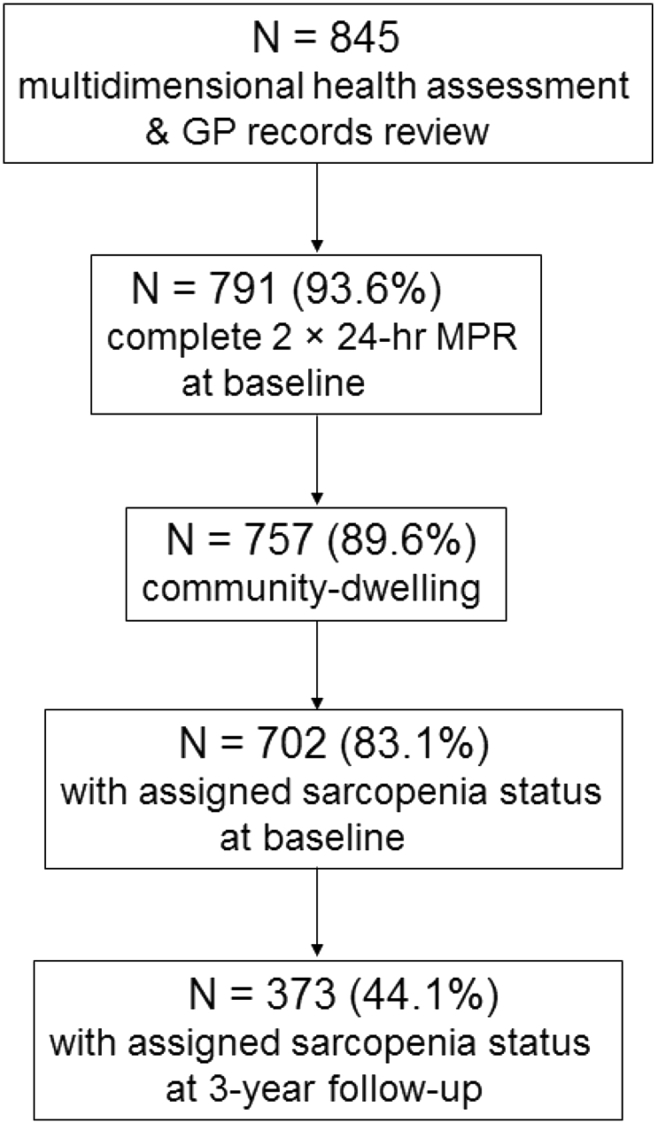
Flowchart of participants in the Newcastle 85 + Study. At baseline, 757 participants (89.6% of sample with complete multidimensional health assessment and GP records review) had dietary assessments (2 × 24-h multiple pass dietary recall) and lived in the community (analytic sample). Of those, 702 (83.1%) had complete data to establish sarcopenia (i.e. grip strength or gait speed, and muscle mass) at baseline and 373 (44.1% of analytic sample) had data at follow-up 3 years later.

The Newcastle 85 + Study is a prospective cohort study of health and functioning of over 1000 participants (birth cohort 1921) who were registered with general practices in Newcastle and North Tyneside, UK. The study has been described in detail previously [45], [46]. Briefly, participants (aged ≥85 years) were assessed using a comprehensive health assessment at baseline (wave 1, 2006/07), and followed at 18 months (1.5 years; wave 2), 36 months (3 years; wave 3), and 60 months (5 years; wave 4) by trained research nurses at their usual place of residence. At baseline, 845 participants had a multidimensional health assessment (including muscle mass, muscle strength, and physical performance) and general practice records review (GPrr), and 757 (463 (61.2%) women) were community-dwelling and had dietary intake data (i.e. analytic sample). Of those, 702 (83.1% of analytic sample) participants had complete data at baseline to establish their sarcopenia status based on the EWGSOP criteria, and 373 (44.1%) participants at 3-year follow-up (wave 3) (Fig. 1).

### 2.2 Ethics statement

The study was approved by the Newcastle & North Tyneside Local Research Ethics Committee 1 and in agreement with The Code of Ethics of the World Medical Association (Declaration of Helsinki). Signed consent was obtained from each participant or, where participants lacked capacity, a signed consultee approval was obtained prior to the study commencement.

### 2.3 Study variables

#### 2.3.1 Dietary assessment

Dietary assessment and validation of 24 h multiple pass dietary recall (24-hr MPR) in the Newcastle 85 + Study have been described previously [47], [48]. Briefly, a pilot study in a sub-sample of this cohort determined that 24-hr MPR was more appropriate for individual dietary assessment of the very old and more accurate in estimating energy and nutrient intake compared with the food frequency questionnaire (FFQ) [47]. At baseline (2006/07), trained research nurses made a detailed record of foods eaten on the previous day (e.g. type, amount and eating occasion) for each participant on two non-consecutive days (except Fridays and Saturdays), at least a week apart. Each food was assigned a unique food code (>2000 codes) and intakes (in g; mean for 2 days) were entered in a Microsoft Access-based dietary data system. Food codes were grouped into 118 food groups (based on McCance and Widdowson's The Composition of Foods [47], [48], [49]), and further combined into 33 food groups (based on food/nutrient composition similarities) as described previously [48]. These were classified as absent (coded 0 if food not consumed) or present (coded 1 if food consumed) for each participant. Thirty food groups were used in the cluster analysis to derive DPs as described [48].

#### 2.3.2 Protein intake categorisation

Low protein intake at baseline was defined as intake of <1 g protein/adjusted (ideal) BW/day (<1 g/kg aBW/d) as described previously [31], [33]. This cut-off was based on previous findings from our group showing that protein intake <1 g/kg aBW/d was associated with lower grip strength (GS) and slower performance on Timed Up-and-Go (TUG) test [31] at baseline, whilst intake of ≥1 g/kg aBW/d was associated with better disability trajectories from 85 to 90 years in this cohort [32]. For g/kg aBW/d calculations, measured body weight was adjusted to a desirable body weight if a participant was outside a healthy body mass index (BMI) range of 22–27 for an older adult aged ≥71 years (described in [50]). The 22–27 BMI range has been associated with a decreased risk of mortality, and led to higher estimates of protein inadequacy in those aged ≥71 years [50]. The aBW was then used to establish protein intake cut-offs (low [<1 g/kg aBW/d] versus good [≥1 g/kg aBW/d]) in 722 participants (of whom 56.1% had BW adjusted to healthy BMI) [31], [32], [33]. As a sensitivity analysis, we re-fitted the models using protein intake dichotomised to < and ≥0.8 g/kg actual BW/d and < and ≥1.0 g/kg actual BW/d cut-offs.

#### 2.3.3 EWGSOP definition of sarcopenia

Prevalence (wave 1 and wave 3) and incidence of sarcopenia (wave 3) in this cohort have been established previously [4] using the EWGSOP definition [1]. Briefly, we used the following criteria and the EWGSOP algorithm: (a) low skeletal muscle index (SMI; skeletal muscle mass divided by height square, kg/m^2^) based on previously established cut-offs (<8.87 kg/m^2^ in men, and <6.67 kg/m^2^ in women) [51], and either (b) slow gait speed (≤0.8 m/s) or (c) weak GS (<16 kg in women, and <26 kg in men) [52].

GS (kg) was measured twice in each hand using a Takei A5401 digital dynamometer (Takei Scientific Instruments Ltd., Niigata, Japan) in the standing position. The maximum GS was used for analyses. To estimate gait speed (m/s), we used the following formula to convert the TUG) test times: 6/[TUG time]) * 1.62. The TUG test measured the time needed to get up from a chair and walk as quickly and safely as possible 3 m in a straight line up to a marked spot on the floor, turn around, walk back and sit back on the chair. Body composition, including muscle mass, was estimated with the Tanita-305 bioimpedance inbuilt algorithm (Tanita Corp., Tokyo, Japan).

#### 2.3.4 Other covariates and potential risk factors of sarcopenia

We considered the following potential risk factors (assessed at baseline) for sarcopenia which were previously identified in this cohort [4], [53] and regarded as clinically important. Socio-demographic factors included: (a) sex; (b) social class (higher managerial and administrative/intermediate/manual and routine occupations) coded to the National Statistics Socio-economic Classification System (NS-SEC), and (c) education (0–9/10–11/≥12 years full-time). Anthropometry and health-related factors were: (a) BMI (kg weight/m^2^ height; underweight [<18.5]/normal [>18.5–25]/overweight and obese [>25]); (b) cognitive status (impaired [<26 points on the Standardised Mini Mental State Examination, SMMSE]/normal [≥26 points on SMMSE]); (c) depressive symptoms (none [score 0–5]/mild or moderate [score 6–7]/severe [score 8–15] assessed by the Geriatric Depression Scale (GDS-15); (d) total number of chronic diseases reported from the GPrr (0–1/2/≥3), and (e) total number of medications (0–2/3–4/≥5). Lifestyle factors included: (a) physical activity (low [score 0–1]/moderate [score 2–6]/high [score 7–18]); (b) smoking (never smoker/current smoker/former smoker), and (c) total energy from foods (continuous; kJ).

Self-reported physical activity was assessed with a purpose-designed physical activity questionnaire which measured the frequency and intensity of highly energetic, moderately energetic, and mildly energetic activities during daily life as described previously [45], [54]. Objectively measured physical activity (actigraphy) correlated well with the physical activity scores in wave 3 [54]. Chronic diseases included cardiovascular disease (hypertension, cardiac disease, cerebrovascular diseases), respiratory diseases, diabetes, arthritis, and cancer [46].

In sensitivity (multivariable) analyses, retention (completed the study/dropped out [by withdrawal or death]) was included as a covariate to account for the ‘healthy survivor effect’. Death data were obtained through the NHS Digital (previously Health and Social Care Information Service UK). BMI (kg/m^2^) was also categorised as <22/22–27/>27 with the middle category as a ‘normal’ BMI for older adults aged ≥71 years [50].

### 2.4 Statistical analysis

#### 2.4.1 Derivation of DP

Derivation of DP have been described in detail previously [48], [53] and summarised in the Supplementary Information (Appendix 1).

#### 2.4.2 Descriptive statistics

Participants were compared on key sociodemographic, anthropometric, health-related, lifestyle, dietary, and nutritional variables by DP using ANOVA (with post-hoc Tukey HDS or Games-Howell) for normally distributed data, Kruskal–Wallis for non-normally distributed and ordinal data, and the Chi-square test for categorical variables.

#### 2.4.3 Multivariable analysis

We fitted several logistic regression models to explore the association between DP and prevalent sarcopenia (at baseline and at 3-year follow-up) and 3-year incident sarcopenia in all participants and when stratified by protein intake (low [<1 g/kg aBW/d] versus good [≥1 g/kg aBW/d]) (OR 95% CI).

Model 1 was unadjusted. Model 2 was adjusted for socio-demographic factors (sex, education, and social class) and BMI (in all participants but not in stratified analysis). Model 3 was additionally adjusted for health-related factors (cognitive status, depressive symptoms, total number of diseases and medication). Model 4 was further adjusted for lifestyle factors (physical activity, smoking, and food energy intake).

#### 2.4.4 Sensitivity analysis

Model 4 was additionally adjusted for retention (completing the study or not) to account for any survivor effect (i.e. healthier and more robust individuals affecting the associations). We observed that participants who completed all four waves of the study had less chronic diseases, including cognitive impairment and sarcopenia, lower intake of medication, higher self-rated health, fewer disabilities, higher muscle strength and walking speed compared with those who were lost to follow-up (details not shown). We also explored whether a protein intake and physical activity interaction term influenced the association between DPs and prevalent sarcopenia in all participants, and repeated the models with re-categorised BMI (22–27 kg/m^2^ as normal/ideal category for older adults aged ≥71 years). Finally, we dichotomised protein intake by 0.8 and 1 g/kg actual BW/day to examine the robustness of the findings obtained in the main analysis with 0.8 and 1 g/kg aBW/d.

We further hypothesised that participants who died between wave 2 (1.5-year follow-up) and wave 3 (3-year follow-up) were at higher risk of sarcopenia (i.e. experiencing terminal decline) than those who were alive. Therefore, in sensitivity analysis, we combined incident cases of sarcopenia at 3-year follow-up with those who died between wave 2 and 3, and repeated the models (in all participants, and stratified by protein intake).

Multicollinearity of covariates was assessed by multicollinearity diagnostics (i.e. Tolerance, Eigenvalues and Condition Index). All analyses were conducted using IBM SPSS (V.21; IBM Corporation, Armonk, NY, USA), and statistics were 2-sided at α = 0.05.

## 3 Results

DPs in the Newcastle 85 + Study participants have been described in detail previously with the entire cohort, including those in care homes [48], [53]. DP differentiation, nutritional, socio-demographic and health characteristics of the present analyses in community-dwelling older adults were very similar to those previously reported in a bigger sample, and they are described in Appendix 1.

### 3.1 Sarcopenia status by dietary patterns

Table 1 describes the prevalence and incidence of sarcopenia by DPs. At baseline, DP2 (‘Traditional British’) was associated with a greater likelihood of sarcopenia compared with other DPs, though this did not reach statistical significance (p = 0.07). Participants belonging to DP3 (‘Low Butter’) were the least likely to have sarcopenia at 3-year follow-up (p = 0.009).

**Table 1:** Sarcopenia status in the Newcastle 85 + Study participants by DPs.

Characteristic	DP1: Low Red Meat	DP2: Traditional British	DP3: Low Butter	p*
	n = 245	n = 231	n = 281	
*Sarcopenia status*				
Sarcopenia (baseline) % (n)				0.07
No	32.1 (179)	27.5 (153)	40.4 (225)	
Yes	31.7 (46)	36.6 (53)	31.7 (46)	
3-year prevalent sarcopenia % (n)				0.009
No	36.3 (107)	24.7 (73)	39.0 (115)	
Yes	34.6 (27)	41.0 (32)	24.4 (19)	
3-year incident sarcopenia % (n)				0.23
No	36.3 (97)	22.8 (61)	40.8 (109)	
Yes	30.3 (10)	36.3 (12)	33.3 (11)	

χ^2^ test for categorical variables.

DPs, dietary patterns.

### 3.2 Dietary patterns, *prevalent* sarcopenia (at baseline and 3-year follow-up) and 3-year *incident* sarcopenia in all participants

At baseline, 145 participants (19.2% of analytic sample) had sarcopenia and 78 were classified as sarcopenic at 3-year follow-up (20.7% of sample available at 3-year follow-up) of whom, 33 were incident cases (i.e. 42.3% of those with sarcopenia at 3-year follow-up were new cases) (details not shown). After adjustment for socio-demographic and health-related factors and BMI (Model 3), participants in DP2 (‘Traditional British’) had increased odds of sarcopenia at baseline (OR = 1.75 [95% CI: 1.06–2.90], p = 0.03) and 3-year follow-up (OR = 2.57, 95% CI: 1.26–5.26, p = 0.01) compared with those in DP3 (‘Low Butter’) (Table 2). Further adjustment for lifestyle factors (Model 4) reduced the odds to non-significant at baseline (OR = 1.64, 0.98–2.77, p = 0.06), but it remained significant for prevalent sarcopenia at 3-year follow-up (OR = 2.42, 1.15–5.09, p = 0.02). The association was not changed by adding the retention variable (completing the study or not) in sensitivity analysis (OR = 2.45, 1.17–5.15, p = 0.02) (details not shown).

**Table 2:** Association between DPs and odds of prevalent sarcopenia (at baseline and 3-year follow-up)^a^ and 3-year incident^b^ sarcopenia (OR, 95% CI) in all participants.

Dietary patterns (n)	Model 1	p	Model 2	p	Model 3	p	Model 4	p
*Sarcopenia (baseline)*								
n	702		657		655		645	
DP1	1.26 (0.80–1.98)	0.32	1.34 (0.80–2.22)	0.27	1.38 (0.82–2.33)	0.23	1.31 (0.77–2.22)	0.32
DP2	1.70 (1.09–2.64)	0.02	1.74 (1.06–2.83)	0.03	1.75 (1.06–2.90)	0.03	1.64 (0.95–2.77)	0.06
DP3 (ref)	1		1		1			1
*3-year prevalent sarcopenia*								
n	373		356		356			353
DP1	1.53 (0.80–2.91)	0.2	1.87 (0.92–3.82)	0.08	1.85 (0.89–3.84)	0.1	1.77 (0.84–3.74)	0.13
DP2	2.65 (1.40–5.03)	0.003	2.72 (1.35–5.46)	0.005	2.57 (1.26–5.26)	0.01	2.42 (1.15–5.09)	0.02
DP3 (ref)	1		1		1			1
*3-year incident sarcopenia*								
n	300		288		288			286
DP1	1.02 (0.42–2.51)	0.96	1.23 (0.46–3.30)	0.68	1.19 (0.43–3.33)	0.73	1.05 (0.37–3.03)	0.92
DP2	1.95 (0.81–4.68)	0.13	1.98 (0.76–5.13)	0.16	1.83 (0.67–5.00)	0.24	1.67 (0.59–4.67)	0.33
DP3 (ref)	1		1		1			1

DP1 ‘Low Red Meat’; DP2 ‘Traditional British’; DP3 ‘Low Butter’.

OR, odds ratios; CI, confidence intervals; DPs, dietary patterns; ref, reference group.

Model 1 is unadjusted.

Model 2 is adjusted for socio-demographic factors (sex, social class, education) and body mass index.

Model 3 is additionally adjusted for health-related factors (cognitive status, depressive symptoms, total number of diseases, and total number of medication).

Model 4 is further adjusted for lifestyle factors (physical activity, smoking, and food energy).

a Sarcopenia status was determined using the European Working Group on Sarcopenia in Older People (EWGSOP) definition as described previously [4].

b Data from two waves (2006/07 to 2009/10) were used for incidence sarcopenia. n indicated the number of participants with complete data (sarcopenia status (yes/no), DP and risk factors).

Neither DP1 nor DP2 was associated with the risk of incident sarcopenia at 3-year follow-up (Table 2).

### 3.3 Dietary patterns, *prevalent* sarcopenia (at baseline and 3-year follow-up) and 3-year *incident* sarcopenia in low (<1 g/kg aBW/d) and good (≥1 g/kg aBW/d) protein intake groups

Similarly, we investigated the relationship between DPs and prevalent and incident sarcopenia in the low (<1 g/kg aBW/d) and good (≥1 g/kg aBW/d) protein intake groups (Table 3). In the low protein intake group (n = 332, 46% of analytic sample), 81 (21.5%) and 34 (18.2%) had prevalent sarcopenia at baseline and 3-year follow-up, respectively, and 14 (9.3%) had incident sarcopenia. In the good protein intake group (n = 390, 54% of analytic sample), 64 (19.8%) and 43 (23.8%) had prevalent sarcopenia (at baseline and 3-year follow-up, respectively), and 19 (12.8%) had incident sarcopenia. The differences in sarcopenia prevalence/incidence between the groups were not significant (p ≥ 0.4) (details not shown).

**Table 3:** Association between DPs and odds of prevalent sarcopenia (at baseline and 3-year follow-up)^a^ and 3-year incident^b^ sarcopenia (OR, 95% CI) in low and good protein intake group.

Low protein intake group (<1 g/kg aBW/day)								
Dietary patterns (n)	Model 1	p	Model 2	p	Model 3	p	Model 4	p
Sarcopenia (baseline)								
n	376		360		356		354	
DP1	1.19 (0.65–2.18)	0.57	1.18 (0.62–2.23)	0.62	1.13 (0.58–2.19)	0.72	1.00 (0.51–1.96)	0.99
DP2	1.64 (0.88–3.07)	0.12	1.49 (0.78–2.86)	0.23	1.47 (0.75–2.89)	0.26	1.20 (0.59–2.42)	0.62
DP3 (ref)	1		1		1		1	
3-year prevalent sarcopenia								
n	187		181		181		179	
DP1	1.53 (0.80–2.91)	0.84	1.27 (0.48–3.42)	0.63	1.03 (0.36–2.93)	0.95	1.00 (0.34–2.90)	0.99
DP2	2.65 (1.40–5.03)	0.16	1.86 (0.70–4.96)	0.21	1.77 (0.64–4.89)	0.27	1.63 (0.56–4.81)	0.37
DP3 (ref)	1		1		1		1	
3-year incident sarcopenia								
n	150		145		145		144	
DP1	0.93 (0.25–3.39)	0.91	1.12 (0.29–4.34)	0.87	0.88 (0.20–3.78)	0.86	0.91 (0.20–4.26)	0.91
DP2	1.25 (0.31–5.00)	0.75	1.29 (0.31–5.43)	0.73	1.39 (0.28–6.84)	0.69	1.39 (0.26–7.49)	0.70
DP3 (ref)	1		1		1		1	
Good protein intake group (≥1 g/kg aBW/day)								

Dietary patterns (n)	Model 1	p	Model 2	p	Model 3	p	Model 4	p
Sarcopenia (baseline)								
n	324		310		302		301	
DP1	1.32 (0.65–2.67)	0.45	1.59 (0.74–3.42)	0.24	1.87 (0.84–4.15)	0.23	1.84 (0.81–4.14)	0.14
DP2	1.75 (0.93–3.31)	0.08	1.80 (0.92–3.51)	0.08	1.93 (0.95–3.91)	0.07	2.14 (1.01–4.53)	0.047
DP3 (ref)	1		1		1		1	
3-year prevalent sarcopenia								
n	181		176		176		175	
DP1	2.16 (0.89–5.27)	0.09	3.32 (1.26–8.74)	0.02	3.30 (1.17–9.29)	0.02	2.94 (0.98–8.87)	0.06
DP2	3.38 (1.40–8.16)	0.007	3.38 (1.36–8.40)	0.009	3.91 (1.48–10.29)	0.006	5.45 (1.81–16.36)	0.003
DP3 (ref)	1		1		1		1	
3-year incident sarcopenia								
n	149		144		144		143	
DP1	1.17 (0.34–4.09)	0.81	1.69 (0.43–6.72)	0.45	1.32 (0.29–6.00)	0.72	0.74 (0.13–4.11)	0.73
DP2	2.71 (0.86–8.55)	0.09	2.52 (0.76–8.29)	0.13	2.45 (0.69–8.73)	0.17	3.44 (0.79–14.91)	0.10
DP3 (ref)	1		1		1		1	

DP1 ‘Low Red Meat’; DP2 ‘Traditional British’; DP3 ‘Low Butter’.

OR, odds ratios; CI, confidence intervals; aBW, adjusted body weight; DPs, dietary patterns; ref, reference group.

Model 1 is unadjusted.

Model 2 is adjusted for socio-demographic factors (sex, social class, education).

Model 3 is additionally adjusted for health-related factors (cognitive status, depressive symptoms, total number of diseases, and total number of medication).

Model 4 is further adjusted for lifestyle factors (physical activity, smoking and food energy).

a Sarcopenia status was determined using the European Working Group on Sarcopenia in Older People (EWGSOP) definition as described previously [4].

b Data from two waves (2006/07 to 2009/10) were used for incidence sarcopenia. n indicated the number of participants with complete data (sarcopenia status (yes/no), DP and risk factors).

DPs were not associated with odds of prevalent and incident sarcopenia in the low protein intake group (Table 3). However, in the good protein intake group, belonging to DP2 (‘Traditional British’) was associated with the increased risk of prevalent sarcopenia (at baseline: OR = 2.14, 95% CI: 1.01–4.53, p = 0.05; 3-year follow-up: OR = 5.45, 1.81–16.39, p = 0.003 compared with DP3 (‘Low Butter’) after adjustment for all covariates. Adding the retention variable in sensitivity analysis did not change the findings (details not shown).

### 3.4 Results for sensitivity analysis

Adding the interaction term between protein intake and physical activity to Model 4 did not attenuate the association between prevalent sarcopenia and DP2 (OR = 2.36, 1.13–4.34, p = 0.02) in all participants (details not shown). Using re-categorised BMI (22–27 kg/m^2^ as a normal category) in the analysis with all participants did not change the findings (e.g. 3-year prevalent sarcopenia: 2.47, 1.17–5.02, p = 0.02) (Appendix 2, Supplementary Table 5).

Neither DP1 nor DP2 was significantly associated with the odds of incident sarcopenia when sarcopenia cases were combined with participants who died 1.5 years before 3-year follow-up (deaths between wave 2 and 3, n = 88) in the fully adjusted models.

Similar to the main results (Table 2), DP2 was not associated with sarcopenia (prevalent or incident) if the low protein intake group was defined as <0.8/g kg BW/d or <1 g/kg BW/d (Appendix 2, Supplementary Tables 6 and 7, respectively). In the good protein intake group defined as ≥0.8 g/kg BW/d, the ORs for the association between DP2 and sarcopenia (at baseline and 3-year follow-up) remained raised, but were no longer significant in the fully adjusted models (1.67, 0.96–2.92, p = 0.7; 2.18, 1.00–3.81, p = 0.054, respectively). However, DP2 was associated with 3-year prevalent sarcopenia in the good protein intake group defined as ≥1 g/kg BW/d (3.62, 1.33–9.88, p = 0.01), confirming the results from Table 2.

## 4 Discussion

In the present study, we investigated the risk of prevalent and incident sarcopenia over 3 years in relation to DPs and protein intake in community-dwelling adults aged ≥85 living in North East England, UK. In a model adjusted for key covariates, DP2 (‘Traditional British’), a group in which a higher proportion of people ate butter, red meats/meat dishes, gravy, potatoes, vegetables, sweets/desserts, and with the highest intake of fat and total energy, was associated with a 2.4-fold increased risk of sarcopenia at 3-year follow-up compared with DP3 (‘Low Butter’), a DP in which more people ate unsaturated fat spreads and oils, and with the highest %E from protein and starch. In addition, DP2 was associated with a 2.1-fold and a 5.4-fold increased risk of prevalent sarcopenia at baseline and 3-year follow-up, respectively in participants with good protein intake (≥1 g/kg aBW/d). DP2 was not associated with incident sarcopenia over 3 years. These results add to a limited literature reporting studies that used a ‘whole diet’ approach to understanding the role of nutrition in sarcopenia in older adults, especially in the very old who are at higher risk of both malnutrition [33], [35], [36] and sarcopenia [3], [4], [5].

Many observational studies have used a ‘single nutrient’ approach (e.g. protein or vitamin D) and elements of sarcopenia (muscle mass, strength and function) to explore diet-muscle health relationships [17], [18], [22], [23], [24], [25], [29], [55] but there are few such studies in very old adults [31]. The value of this approach in nutritional research for sarcopenia is well established [56], but overlooks the interactions between food groups and nutrients within diets, and the likely complex, cumulative, synergistic and antagonistic influences of various nutrients/foods on aged muscle. Recent consensus papers on the definition of sarcopenia [1], [2] and the recognition that sarcopenia is a major health problem that contributes, fundamentally, to functional impairment and decline in older adults, have stimulated interest in understanding the role of modifiable factors such as (whole) diet in the aetiology, prevention and management of sarcopenia. Several studies have used a ‘whole diet’ approach and utilised either (a) pre-defined dietary indices (based on the current knowledge about DPs that are associated with better health) or (b) data-driven approaches using statistical techniques to derive factors or clusters without any prior hypotheses about diet-muscle function relationships to investigate the impact of DPs on elements of sarcopenia [43], [44], [57], [58], [59], [60]. In all, higher adherence to a DP described as ‘Prudent diet’ (i.e. higher intake of foods beneficial for health such as fish, fruits and vegetables) or Mediterranean-style diet (MED) was associated with stronger grip, faster walking speed [57], [58], and slower mobility decline over 8–9 years in older adults aged 65 and over [59], [60]. Using data from the Newcastle 85 + Study, we have reported recently that a DP high in fruits, fish, eggs, nuts, and whole grains (but low in red/processed meats and potatoes) was associated with stronger grip and faster TUG in very old adults compared with DPs high in butter and red/processed meats [53].

We are aware of only a few studies [40], [41], [42]; and reviewed in [43], [44] that used a ‘whole diet’ approach to investigate the risk of sarcopenia in adults aged ≥55, and none in the very old. This limits the opportunity for direct comparisons of our findings with those from other studies. All studies defined sarcopenia as loss of muscle mass, and strength or function (i.e. using the EWGSOP or the Asian Working Group for Sarcopenia algorithm), and derived DPs based on FFQ [40], [41], [42]. In all studies, higher adherence to a healthy DP such as MED [40], [42], Baltic Sea Diet (BSD) [42], ‘vegetable-fruits’ DP and better diet quality (Diet Quality Index-International; DQI-I) [41] was associated with lower risk of sarcopenia. Specifically, the highest quartile of BSD (dietary index developed to account for ‘beneficial’ foods consumed routinely in the Nordic countries, such as berries, salmon, rapeseed oil and dairy) was associated with a 67% lower risk of sarcopenia over 3-year follow-up in older women [42]. Asian older men with the highest diet quality had a 50% lower risk of sarcopenia, and those in the highest ‘vegetable-fruit’ DP had a 40% reduced risk [41] compared with those with lower adherence scores. In the present study, membership of DP2 (‘Traditional British’) characterised by a higher proportion of participants consuming butter, red meats, gravy, potatoes, and sweets/desserts substantially increased the risk of prevalent sarcopenia (by 2–5-fold), compared with participants in DP3 (‘Low Butter’). This effect was apparent even in those with good protein intake (≥1 g/kg aBW/d or ≥1 g/kg BW/d). DP3 had some elements of MED such as the highest percentage (91%) of participants consuming unsaturated fat spreads and oils (olive oil and other plant-based fats), and the lowest intakes of SFA, cholesterol and %E from fat but the highest %E from protein and starch, and the highest intake of fibre. These dietary characteristics, in combination with high to moderate intake of other foods (red meat, soups, vegetable, and legumes; Supplementary Table 1 and Supplementary Table 2) and lower intake of less healthy foods (saturated fats spreads, gravy, and sweets/desserts) may ameliorate progressive loss of muscle mass and strength in the very old.

Multiple biological mechanism may have contributed to the increased risk of sarcopenia in participants consuming the ‘Traditional British Diet’ (DP2), including the type and quality of dietary fat consumed within DPs. Although the pathophysiology of sarcopenia is complex and not completely explained, several processes have been recognised to contribute to muscle wasting and loss of function. These include (a) inflammation and production of pro-inflammatory cytokines [61]; (b) imbalance between muscle protein anabolism and catabolism, which may be due to anabolic resistance [62], and (c) inter- and intramyocellular lipid accumulation [63] affecting the quality of the aged muscle.

Dietary fats have a central role in muscle metabolism as an important source of energy [64]; they are an integral part of myocellular membranes [65], and have been shown to affect muscle protein synthesis (MPS) [66]. The composition and amount of dietary fats influence inflammation [67] and insulin resistance [68]—both mechanisms linked to sarcopenia. DP2 (compared to DP3) had less favourable fatty acid intake and composition (e.g. the highest intake of SFA, the lowest MUFA/SFA ratio), which may have exacerbated pro-catabolic processes (i.e. inflammation, insulin resistance, oxidative stress) and increased fat deposition in the aged muscle.

Participants in DP3 (‘Low Butter’) had the highest %E from protein and starch, whilst those in DP2 (‘Traditional British’) had the highest %E from fat (SFA). However, it is still debatable whether higher food intake and excess energy (quantity) or source of energy (quality) is independently associated with sarcopenia (reviewed in [56]). To date, there is a limited evidence about a direct relationship between protein intake and sarcopenia (discussed in [17]), however evidence is also emerging that supports the importance of higher protein intake in combination with exercise (physical activity) to combat anabolic resistance (and stimulate MPS)—one of the mechanisms implicated in sarcopenia in older adults [19], [22], [29]. Our results suggest that good protein intake may not be sufficient to protect against sarcopenia if the combination of other foods in diet are not favourable. Although the participants in DP2 (‘Traditional British’) were the least physically active, the main effect of DP2 on the risk of sarcopenia remained independent after adjustment for protein intake and physical activity interaction term.

We did not observe any association between DPs and incident sarcopenia even when we combined participants who died 1.5 years before sarcopenia follow-up (wave 3) with incident sarcopenia cases. Our study may have not been sufficiently powered to detect such an association, and because of higher prevalence of robust ‘survivors’ and pre-sarcopenia cases who did not transition to sarcopenia during the period of study. In addition, participants who died between baseline and follow-up may have done so because of other health problems not related to sarcopenia.

This study has several limitations that need to be considered when interpreting the results. The labels given to DPs may not represent the most important (food) component or biological aspect of the DP, and low or no intake of foods/nutrients may be as important as those foods that were consumed more frequently or in larger amounts in relation to sarcopenia. The outcomes of cluster analysis are dependent on the specific food groupings used and the decisions taken when deriving such grouping may have influenced the final DPs. Diet was assessed at baseline only and no information about diet change over 3 years was available, although there is some evidence of diet stability across the life course among British older adults [69]. Other diet-related factors such as appetite loss, social support, dietary knowledge, and access to food may have affected participants’ food choices, and may have contributed to Type I error in the logistic regression models. The lack of power in data (especially in stratified analysis) may have resulted in Type II error. The results may not be generalisable to other populations of very old people with e.g. different ethnic backgrounds. The study has several strengths, including its prospective design (a 3-year follow-up for sarcopenia), validated dietary assessment [47], adjustment for several known factors associated with sarcopenia [3], [4], and robustness of the results explored in sensitivity analysis.

In summary, using a data-driven approach and reported dietary intakes in community-dwelling older adults, we derived three DPs that were associated with several health characteristics including sarcopenia. Participants in the DP2 group (‘Traditional British’), a diet high (i.e. high proportion of consumers) in butter, red meats/meat dishes, gravy, potatoes, vegetables, sweets/desserts, and the highest intake of fat and total energy had an increased risk of sarcopenia regardless of protein status compared with DP3 (‘Low Butter’), a diet high in unsaturated fat spreads/oils, fibre, and the highest %E from protein and starch. Findings from this study need to be replicated in other cohorts of the very old to understand the role of dietary patterns in sarcopenia onset and management in populations at risk of functional decline and dependence.

## Statement of authorship

AG designed the research. KD and CJ were responsible for the Newcastle 85 + study design, management and data acquisition. AG analysed data and wrote the manuscript. AAS, NM, TRH, KD, MS, JCM, and CJ revised the manuscript for important intellectual content. AG had primary responsibility for final content. All authors read and approved the version submitted.

## Conflicts of interest

None declared.

## Funding

Funding for this research is provided by the European Horizon 2020 PROMISS Project ‘Prevention Of Malnutrition In Senior Subjects in the EU’, Grant agreement no. 678732 (AG, NM, CJ). The content only reflects the author's view and the Commission is not responsible for any use that may be made of the information it contains. The research was also supported by the National Institute for Health Research Newcastle Biomedical Research Centre, based at Newcastle upon Tyne Hospitals NHS Foundation Trust and Newcastle University (AG, AAS).

The core Newcastle 85 + study was supported by a joint grant from the UK Medical Research Council and the Biotechnology and Biological Sciences Research Council (grant reference G0500997), the Dunhill Medical Trust (grant reference R124/0509), the Medical Council Research grant (G0601333), and NHS North of Tyne (Newcastle Primary Care Trust). Funding sources had no role in the collection, analysis and interpretation of data, the writing, and the decision to submit this article for publication.

## Footnotes

Appendix A Supplementary data to this article can be found online at https://doi.org/10.1016/j.clnu.2019.01.009.
