# Supplementary material for: Effects of dietary patterns and low protein intake on sarcopenia risk in the very old: The Newcastle 85+ study
Source: Clin Nutr. 2020 Jan;39(1):166–73. doi: 10.1016/j.clnu.2019.01.009 (PMC6961212; doi:10.1016/j.clnu.2019.01.009)
Supplement: Multimedia component 1 [file mmc1.docx]

**SUPPLEMENATRY INFORMATION**

**APPENDIX 1.**

**Derivation and characteristics of dietary patterns (DPs) in community-dwelling older adults in the Newcastle 85+ Study**

*Statistical analysis: Derivation of dietary patterns*

We used two-step clustering to derive DPs as described [48]. Briefly, the procedure uses a log-likelihood distance criterion to create small pre-clusters (step 1) and agglomerative hierarchical clustering to merge them into distinct dietary groups (step 2). The best cluster solution was determined by automatic selection and the Bayesian Information Criterion (BIC) with 30 good groups, and the robustness and stability of the DP solution was re-examined by random ordering of cases and by comparing DP characteristics [48].

*Results: Dietary patterns characteristics*

Ten food groups with the highest importance factor (IF) contributed the most to a three-cluster solution (Supplementary Table 1, Supplementary Figure 1). The ‘Low Red Meat’ group (DP1; n=245, 32.4%) had the lowest percentage of participants eating legumes (21.6%), sweets and desserts (53.3%), and the highest percentage of those consuming fish/sea food (58.4%). The ‘Traditional British’ group (DP2, n=231, 30.5%) had the highest percentage of participants consuming butter (80.5%), red meats and meat dishes (85.3%), gravy (61.9%), potatoes and potato dishes (96.1%), vegetables (95.2%), and sweets/desserts (74.9%). The ‘Low Butter’ group (DP3, n=281, 37.1%), the largest cluster, had the highest percentage of participants eating unsaturated fat spreads and oils (91.1%). The percentages of participants consuming 10 food groups paralleled the mean daily intake (g/day) of these foods across DPs (Supplementary Table 2). For example, DP1 had the lowest consumption (g/day) of red meat, potatoes, vegetable and legumes, whilst DP2 had the lowest intake of unsaturated fat spreads and oils, but the highest intake of sweets and desserts (all p<0.001). In all, these DPs were very similar to DPs obtained previously using all participants in the Newcastle 85+ Study with complete diet data (n=793) and including those in care homes [48,53].

*Sociodemographic and health characteristics of dietary patterns*

Compared with other DPs, participants in DP1 (‘Low Red Meat’) were more likely to be women (p=0.01), more educated (p<0.001), and to belong to a higher social class (p=0.002) (Supplementary Table 3). Those in DP2 (‘Traditional British’) were the least physically active (p=0.008). Participants did not differ between DPs in respect of other characteristics.

*Nutritional characteristics of dietary patterns*

DPs varied by several nutritional characteristics and macronutrient intake such as protein, fat and starch (Supplementary Table 4). Compared with DP2 and DP3, DP1 (‘Low Red Meat’) had the lowest intakes of food weight, food energy, carbohydrate, starch, protein (g/day) (all p<0.001) and water (p=0.004). Participants belonging to DP2 (‘Traditional British’) had the highest intake of total energy (from foods, drinks and alcohol), fat, and the lowest monounsaturated fatty acids/saturated fatty acids (MUFA/SFA) ratio compared with other DPs (all p<0.001). Participants in DP3 (‘Low Butter’) had the lowest percent energy (%E) from fat (p<0.001), but the highest %E from protein (p<0.001) and starch (p=0.006), the lowest consumption of SFA and cholesterol, and the highest intake of polyunsaturated fatty acids (PUFA) (g) and fibre (all p<0.001) (Supplementary Table 3). Based on the characteristics and because of lower percentage of participants with prevalent sarcopenia at baseline and 3-year follow-up compared with other DPs, DP3 was considered as potentially the healthiest for muscle health and used as the reference group in multivariate analyses.

**Supplementary Figures**

**
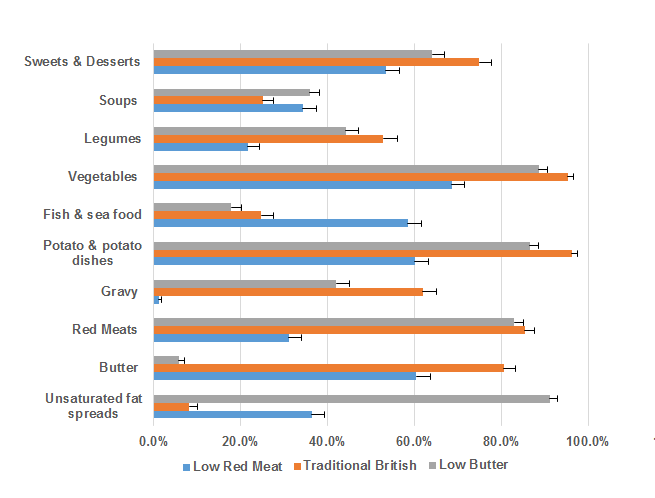
**

**Supplementary Figure 1.** Percentage of participants consuming each food group by dietary patterns (DPs). Ten food groups contributed the most to cluster separation into three distinct DPs: DP1 (‘Low Red Meat’, 32.4% participants; blue bars), DP2 (‘Traditional British’, 30.5% participants; orange bars), and DP3 (‘Low Butter’, 37.1% participants; grey bars). X-axes indicates percentages of participants consuming each food group represented on Y-axes in the order of importance for cluster separation (from bottom to top). Error bars represent standard error of the mean (SEM).

**Supplementary Tables**

**Supplementary Table 1**. Percentage (%) of participants consuming each of 10 food groups with the highest to lowest importance factor (IF) by dietary patterns (DPs)^a^

| Food group | DP1: Low Red Meat | DP2: Traditional British | DP3: Low Butter | IF range: 0-1 |
| --- | --- | --- | --- | --- |
|  | n = 245 (32.4%) | n = 231 (30.5%) | n = 281 (37.1%) |  |
| Unsaturated fat spreads & oils | 36.3 | 8.2 | 91.1 | 1 |
| Butter | 60.4 | 80.5 | 5.7 | 0.85 |
| Red meats & meat dishes | 31.0 | 85.3 | 82.9 | 0.57 |
| Gravy | 1.1 | 61.9 | 42.0 | 0.55 |
| Potato & potato dishes | 60.0 | 96.1 | 86.5 | 0.30 |
| Fish & sea food | 58.4 | 24.7 | 17.8 | 0.29 |
| Vegetables | 68.6 | 95.2 | 88.6 | 0.19 |
| Legumes | 21.6 | 52.8 | 44.1 | 0.14 |
| Soups | 34.3 | 25.1 | 35.9 | 0.07 |
| Sweets & desserts | 53.3 | 74.9 | 64.1 | 0.06 |

^a^DPs were derived using two-step clustering of 30 food groups [48,53]. Ten food groups contributed the most to DP separation. The remainder food groups (saturated fat spreads & margarine, coffee, processed & other meats, low fat dairy, poultry, high fat dairy, soft sugary drinks, eggs, bacon & ham, tea, chocolate, snacks & savouries, nuts, biscuits & cakes, fruits, refined grains & cereal products, whole grains & cereal products, hot drinks, preserves & syrups, and alcohol) had an IF <0.06.

**Supplementary Table 2**. Mean daily intake (g) of 10 food groups contributing the most to DP separation

| Food group | DP1: Low Red Meat | DP2: Traditional British | DP3: Low Butter | p^a^ |
| --- | --- | --- | --- | --- |
| M (SD) | n = 245 | n = 231 | n = 281 |  |
| Unsaturated fat spreads | 6.6 (11.8) | 1.38 (5.8) | 17.3 (15.1) | <0.001 |
| Butter | 11.9 (15.9) | 15.8 (16.5) | 0.7 (3.4) | <0.001 |
| Red meats & meat dishes | 41.54 (82.9) | 81.8 (67.8) | 95.1 (88.0) | <0.001 |
| Gravy | 0.5 (5.2) | 22.6 (30.6) | 20.4 (33.2) | <0.001 |
| Potato & potato dishes | 58.1 (68.93) | 119.4 (80.3) | 106.0 (79.2) | <0.001 |
| Fish & sea food | 40.4 (51.0) | 13.5 (30.8) | 12.0 (32.6) | <0.001 |
| Vegetables | 66.3 (78.9) | 102.5 (70.0) | 101.1 (89.5) | <0.001 |
| Legumes | 10.4 (26.9) | 22.1 (30.2) | 18.8 (29.5) | <0.001 |
| Soups | 60.6 (101.2) | 25.8 (69.1) | 27.1 (64.5) | <0.001 |
| Sweets & desserts | 40.6 (57.5) | 74.8 (78.6) | 58.0 (68.1) | <0.001 |

^a^Kruskal-Wallis test for non-normally distributed data. DP, dietary pattern.

**Supplementary Table 3.** Baseline characteristics of the Newcastle 85+ Study participants by DPs

| Characteristics | DP1:  Low Red Meat | DP2:  Traditional British | DP3:  Low Butter | p^a^ |
| --- | --- | --- | --- | --- |
|  | n = 245 | n = 231 | n = 281 |  |
| *Socio-demographic factors* | | | | |
| Women % (n) | 36.3 (168) | 29.6 (137) | 34.1 (158) | 0.01 |
| Occupational class |  |  |  | 0.002 |
| Routine/manual | 27.1 (99) | 35.9 (131) | 37.0 (135) |  |
| Intermediate | 31.8 (34) | 22.4 (24) | 45.8 (49) |  |
| Higher managerial/ | 39.5 (100) | 26.1 (66) | 34.4 (87) |  |
| administrative professions |  |  |  |  |
| Years of education % (n) |  |  |  | <0.001 |
| 0-9 | 27.0 (130) | 32.0 (154) | 41.0 (197) |  |
| 10-11 | 35.8 (64) | 30.7 (55) | 33.5 (60) |  |
| ≥12 | 53.8 (50) | 21.5 (20) | 24.7 (23) |  |
| *Anthropometry* |  |  |  |  |
| BMI % (n) |  |  |  | 0.72 |
| Underweight (<18.5) | 37.0 (17) | 21.7 (10) | 41.1 (19) |  |
| Normal (>18.5-25) | 32.4 (120) | 30.8 (114) | 36.8 (136) |  |
| Overweigh & obese (>25) | 31.1 (95) | 29.2 (89) | 39.7 (121) |  |
| *Health-related variables* | | | | |
| Number of chronic diseases % (n) |  |  |  | 0.24 |
| 0-1 | 35.5 (79) | 32.9 (73) | 31.5 (70) |  |
| 2 | 29.4 (67) | 32.0 (73) | 38.6 (88) |  |
| ≥3 | 32.2 (99) | 27.7 (85) | 40.1 (123) |  |
| Total number of medication % (n) |  |  |  | 0.08 |
| 0-2 | 36.9 (48) | 34.6 (45) | 28.5 (37) |  |
| 3-4 | 38.1 (48) | 27.0 (34) | 34.9 (44) |  |
| ≥5 | 29.7 (149) | 30.3 (152) | 39.9 (200) |  |
| Depressive symptoms % (n) |  |  |  | 0.69 |
| 0-5/none | 32.8 (192) | 29.0 (170) | 38.2 (224) |  |
| 6-7/mild | 37.2 (35) | 30.9 (29) | 31.9 (22) |  |
| ≥8/severe | 29.3 (17) | 32.8 (19) | 37.9 (22) |  |
| Cognitive status % (n) |  |  |  | 0.09 |
| Impaired (<26 SMMSE score) | 33.9 (194) | 28.4 (163) | 37.7 (216) |  |
| Normal | 27.9 (51) | 36.6 (67) | 35.5 (65) |  |
| *Lifestyle* |  |  |  |  |
| Physical activity % (n) |  |  |  | 0.008 |
| Low | 31.3 (46) | 42.2 (62) | 26.5 (39) |  |
| Moderate | 33.6 (114) | 27.4 (93) | 38.9 (132) |  |
| High | 31.5 (85) | 28.1 (76) | 40.4 (109) |  |
| Smoking |  |  |  | 0.08 |
| Never smoker | 34.6 (91) | 26.2 (69) | 39.2 (103) |  |
| Current smoker | 31.8 (14) | 45.5 (20) | 22.7 (10) |  |
| Former smoker | 31.2 (140) | 31.4 (141) | 37.4 (168) |  |

^a^Kruskal-Wallis test for ordered and non-normally distributed continuous variables and χ^2^ test for categorical variables.

**Supplementary Table 4**. Daily intake of selected nutrients and energy by DP.

| Nutrient / energy, M (SD)^a^ | DP1: Low Red Meat | DP2: Traditional British | DP3: Low Butter | p^b^ |
| --- | --- | --- | --- | --- |
|  | n = 245 | n = 231 | n = 281 |  |
| Total energy (KJ) | 6506 (1998) | 7542 (2165) | 7077.01 (2124) | <0.001 |
| Food energy (KJ) | 6361.76 (1887.64) | 7229.9 (2095.69) | 6924.57 (2106.55) | <0.001 |
| Food weight (g) | 2227.26 (632.49) | 2410.78 (652.50) | 2411.32 (600.9) | 0.001 |
| Food energy density (KJ/g) | 2.94 (0.83) | 3.1 (0.71) | 2.91 (0.68) | 0.01 |
| Fat (g) | 64.84 (26.40) | 77.09 (27.69) | 66.16 (25.0) | <0.001 |
| % Energy from fat | 37.23 (7.22) | 38.58 (6.96) | 34.96 (6.53) | <0.001 |
| Protein (g) | 57.94 (20.2) | 64.93 (21.36) | 68.38 (23.81) | <0.001 |
| % Energy from protein | 15.72 (3.48) | 15.25 (3.44) | 16.82 (3.83) | <0.001 |
| Carbohydrates (g) | 183.48 (56.33) | 209.61 (65.95) | 206.26 (65.4) | <0.001 |
| % Energy from carbohydrates | 46.96 (7.33) | 46.02 (6.81) | 48.15 (6.37) | 0.002 |
| Starch (g) | 95.58 (33.43) | 109.34 (36.72) | 109.89 (39.34) | <0.001 |
| % Energy from starch | 24.5 (6.0) | 24.22 (5.37) | 25.7 (5.56) | 0.006 |
| NMES (g) | 43.46 (27.54) | 53.08 (34.52) | 47.21 (30.11) | 0.01 |
| % Energy from NMES | 10.85 (5.98) | 11.33 (6.18) | 10.76 (5.57) | 0.69 |
| Total sugars (g) | 85.85 (37.11) | 96.68 (42.82) | 93.48 (39.70) | 0.009 |
| % Energy from total sugars | 21.82 (7.26) | 20.96 (6.72) | 21.61 (6.44) | 0.36 |
| NSP (Englyst method^c^) (g) | 10.04 (5.24) | 10.91 (4.27) | 12.04 (5.49) | <0.001 |
| Cholesterol (mg) | 204.43 (127.77) | 226.35 (132.75) | 172.67 (101.39) | <0.001 |
| MUFA (g) | 16.36 (8.05) | 17.96 (8.61) | 16.41 (7.78) | 0.04 |
| PUFA (g) | 6.90 (4.84) | 6.12 (4.01) | 9.26 (5.43) | <0.001 |
| SFA (g) | 25.72 (13.13) | 31.31 (13.70) | 22.84 (10.38) | <0.001 |
| MUFA/SFA ratio | 0.67 (0.25) | 0.58 (0.17) | 0.75 (0.23) | <0.001 |
| Alcohol (g) | 6.19 (12.77) | 7.63 (14.77) | 5.69 (12.6) | 0.12 |
| Water (g) | 1851.08 (565.68) | 1990.6 (580.08) | 1998.88 (526.9) | 0.004 |

^a^The estimates of nutrients were based on consumption of 118 food groups, which were coded by McCance and Widowson’s food composition tables 6^th^ edition [49]. ^b^ANOVA (with post-hoc Tukey HDS or Games-Howell) for normally, and Kruskal-Wallis for non-normally distributed data. ^c^The method measures NSP (fibre) and was the most commonly used method in the UK.

DP, dietary pattern; KJ, kilojoules; NMES, non-milk extrinsic sugars; NSP, non-starch polysaccharides; MUFA, monounsaturated fatty acids; PUFA, polyunsaturated fatty acids; SFA, saturated fatty acids.

**APPENDIX 2.**

**Sensitivity analyses**

**Supplementary Table 5.** Association between DPs and odds of prevalent sarcopenia (at baseline and 3-year follow-up) and 3-year incident sarcopenia (OR 95% CI) in all participants

Body mass index (BMI) was re-categorised with 22-27 kg/m^2^ category as ‘normal’ to test the robustness of the results obtained with BMI categorised as underweight (<18.5) / normal (>18.5-25) / overweight and obese (>25) (Table 2).

**Supplementary Table 6.** Association between DPs and odds of prevalent sarcopenia (at baseline and 3-year follow-up) and 3-year incident sarcopenia (OR 95% CI) in low (<0.8 g/kg BW/d) and good (≥0.8g/kg BW/d) protein intake groups

Protein intake was dichotomised by 0.8 and 1.0g/ actual body weight (BW)/day to compare with the results obtained using 0.8 and 1g/ kg adjusted body weight (aBW)/day, and to test whether using actual versus adjusted BW (normal: 22 to 27 for an older adult aged ≥71 years (described in [50]) have an effect on the findings. Two hundred and six (28.5%) participants had protein intake <0.8g/kg BW/d, and 516 (71.5%) consumed ≥0.8g/kg BW/d of protein.

**Supplementary Table 7.** Association between DPs and odds of prevalent sarcopenia (at baseline and 3-year follow-up) and 3-year incident sarcopenia (OR 95% CI) in low (<1 g/kg BW/d) and good (≥1g/kg BW/d) protein intake group

Protein intake was categorised by 1g/ BW/d cut-off to explore the robustness of the results obtained using 1g/ kg aBW/day (Table 3). Three hundred and sixty-six (46.0%) had protein intake <1g/kg BW/day, and 390 (54.0%) consumed ≥1g/kg BW/d of protein.

**Supplementary Table 5**. Association between DPs and odds of prevalent sarcopenia (at baseline and 3-year follow-up)^a^ and 3-year incident^b^ sarcopenia (OR, 95% CI) in all participants

Dietary patterns (n) Model 1 p Model 2 p Model 3 p Model 4 p

*Sarcopenia (baseline)*

n 702 657 655 645

DP1 1.26 (0.80-1.98) 0.32 1.30 (0.78-2.17) 0.32 1.35 (0.80-2.28) 0.23 1.27 (0.74-2.16) 0.39

DP2 1.70 (1.09-2.64) 0.02 1.61 (0.98-2.63) 0.06 1.75 (0.98-2.72) 0.06 1.54 (0.91-2.62) 0.11

DP3 (ref) 1 1 1 1

*3-year* *prevalent sarcopenia*

n 373 356 356 353

DP1 1.53 (0.80-2.91) 0.2 2.02 (0.99-4.12) 0.054 2.06 (0.99-4.29) 0.053 1.98 (0.94-4.20) 0.07

DP2 2.65 (1.40-5.03) 0.003 2.57 (1.28-5.18) 0.008 2.55 (1.25-5.20) 0.01 2.47 (1.17-5.20) 0.02

DP3 (ref) 1 1 1 1

*3-year* *incident sarcopenia*

n 300 288 288 286

DP1 1.02 (0.42-2.51) 0.96 1.39 (0.52-3.70) 0.51 1.35 (0.49-3.73) 0.56 1.21 (0.43-3.43) 0.92

DP2 1.95 (0.81-4.68) 0.13 1.86 (0.72-4.76) 0.20 1.82 (0.68-4.86) 0.23 1.78 (0.65-4.85) 0.26

DP3 (ref) 1 1 1 1

DP1 ‘Low Red Meat’; DP2 ‘Traditional British’; DP3 ‘Low Butter’.

^a^Sarcopenia status was determined using the European Working Group on Sarcopenia in Older People (EWGSOP) definition as described previously [4]. ^b^Data from two waves (2006/07 to 2009/10) were used for incidence sarcopenia. n indicated the number of participants with complete data (sarcopenia status (yes/no), DP and risk factors).

OR, odds ratios; CI, confidence intervals; DPs, dietary patterns; ref, reference group

Model 1 is unadjusted.

Model 2 is adjusted for socio-demographic factors (sex, social class, education) and re-categorised BMI (<22 kg/m^2^ / 22-27 / >27).

Model 3 is additionally adjusted for health-related factors (cognitive status, depressive symptoms, total number of diseases, and total number of medication).

Model 4 is further adjusted for lifestyle factors (physical activity, smoking, and food energy).

**Supplementary Table 6**. Association between DPs and odds of prevalent sarcopenia (at baseline and 3-year follow-up)^a^ and 3-year incident^b^ sarcopenia (OR, 95% CI) in the low (<0.8 g/kg BW/d) and good (≥0.8g/kg BW/d) protein intake group

*Low protein intake group (<0.8g/kg BW/d)*

Dietary patterns (n) Model 1 p Model 2 p Model 3 p Model 4 p

*Sarcopenia (baseline)*

n 200 191 191 189

DP1 1.29 (0.51-3.28) 0.57 0.97 (0.36-2.64) 0.96 1.02 (0.36-2.91) 0.97 0.75 (0.25-2.29) 0.61

DP2 1.93 (0.73-5.06) 0.18 1.42 (0.50-4.03) 0.51 1.49 (0.51-4.36) 0.47 1.21 (0.39-3.82) 0.74

DP3 (ref) 1 1 1 1

*3-year* *prevalent sarcopenia*

n 97 94 94 92

DP1 1.88 (0.43-8.18) 0.84 1.85 (0.37-9.28) 0.63 3.15 (0.40-25.14) 0.28 2.66 (0.31-22.90) 0.37

DP2 4.44 (1.04-18.95) 0.04 3.67 (0.80-16.84) 0.09 5.53 (0.86-35.68) 0.07 3.84 (0.55-26.81) 0.18

DP3 (ref) 1 1 1 1

*3-year incident sarcopenia*

n 77 75 75 74

DP1 1.12 (0.15-8.55) 0.91 1.09 (0.13-9.38) 0.94 1.21 (0.08-17.70) 0.89 2.46 (0.12-50.26) 0.56

DP2 2.47 (0.37-16.32) 0.35 1.88 (0.26-13.76) 0.53 2.98 (0.24-36.91) 0.40 4.00 (0.26-61.81) 0.32

DP3 (ref) 1 1 1 1

*Good protein intake group (≥0.8g/kg BW/d)*

Dietary patterns (n) Model 1 p Model 2 p Model 3 p Model 4 p

*Sarcopenia (baseline)*

n 500 479 467 466

DP1 1.32 (0.78-2.26) 0.30 1.53 (0.87-2.67) 0.14 1.55 (0.87-2.77) 0.23 1.50 (0.83-2.69) 0.18

DP2 1.64 (0.99-2.72) 0.053 1.64 (0.97-2.75) 0.06 1.65 (0.96-2.84) 0.07 1.67 (0.96-2.92) 0.07

DP3 (ref) 1 1 1 1

*3-year* *prevalent sarcopenia*

n 271 263 263 262

DP1 1.49 (0.72-3.07) 0.28 2.22 (1.02-4.83) 0.045 2.13 (0.95-4.76) 0.07 2.02 (0.89-4.59) 0.09

DP2 2.28 (1.10-4.70) 0.03 2.23 (1.06-4.72) 0.04 2.15 (1.00-4.65) 0.051 2.18 (1.00-4.81) 0.054

DP3 (ref) 1 1 1

*3-year incident sarcopenia*

n 222 214 214 213

DP1 1.01 (0.37-2.77) 0.98 1.42 (0.48-4.19) 0.53 1.46 (0.46-4.59) 0.52 1.31 (0.40-4.30) 0.66

DP2 1.84 (0.68-4.98) 0.23 1.70 (0.61-4.74) 0.31 1.78 (0.60-5.23) 0.30 1.71 (0.57-5.12) 0.34

DP3 (ref) 1 1 1 1

DP1 ‘Low Red Meat’; DP2 ‘Traditional British’; DP3 ‘Low Butter’.

^a^Sarcopenia status was determined using the European Working Group on Sarcopenia in Older People (EWGSOP) definition as described previously [4]. ^b^Data from two waves (2006/07 to 2009/10) were used for incidence sarcopenia. n indicated the number of participants with complete data (sarcopenia status (yes/no), DP and risk factors).

OR, odds ratios; CI, confidence intervals; DPs, dietary patterns; ref, reference group; BW, body weight

Model 1 is unadjusted.

Model 2 is adjusted for socio-demographic factors (sex, social class, education).

Model 3 is additionally adjusted for health-related factors (cognitive status, depressive symptoms, total number of diseases, and total number of medication).

Model 4 is further adjusted for lifestyle factors (physical activity, smoking and food energy).

**Supplementary Table 7**. Association between DPs and odds of prevalent sarcopenia (at baseline and 3-year follow-up)^a^ and 3-year incident^b^ sarcopenia (OR, 95% CI) in the low (<1g/kg BW/d) and good (≥1g/kg BW/d) protein intake group

*Low protein intake group (<1g/kg BW/d)*

Dietary patterns (n) Model 1 p Model 2 p Model 3 p Model 4 p

*Sarcopenia (baseline)*

n 353 336 334 322

DP1 1.24 (0.61-2.54) 0.55 1.08 (0.50-2.31) 0.85 1.11 (0.50-2.47) 0.80 1.00 (0.51-1.96) 0.76

DP2 2.15 (1.06-4.37) 0.04 1.99 (0.95-4.17) 0.07 2.10 (0.96-4.58) 0.06 1.64 (0.72-3.73) 0.24

DP3 (ref) 1 1 1 1

*3-year* *prevalent sarcopenia*

n 183 176 176 174

DP1 1.79 (0.58-5.47) 0.31 2.17 (0.67-7.06) 0.20 1.91 (0.55-6.64) 0.31 1.90 (0.53-6.85) 0.32

DP2 3.56 (1.17-10.83) 0.03 3.29 (1.06-10.27) 0.04 3.52 (1.07-11.53) 0.04 2.94 (0.86-10.11) 0.09

DP3 (ref) 1 1 1 1

*3-year incident sarcopenia*

n 150 144 144 143

DP1 1.19 (0.25-5.58) 0.83 1.61 (0.32-8.05) 0.56 1.27 (0.24-6.81) 0.78 1.54 (0.28-8.46) 0.62

DP2 2.60 (0.58-11.65) 0.21 2.51 (0.54-11.63) 0.24 2.69 (0.53-13.58) 0.23 2.49 (0.46-13.64) 0.29

DP3 (ref) 1 1 1 1

*Good protein intake group (≥1g/kg BW/d)*

Dietary patterns (n) Model 1 p Model 2 p Model 3 p Model 4 p

*Sarcopenia (baseline)*

n 345 332 322 301

DP1 1.56 (0.85-2.87) 0.15 1.59 (0.74-3.42) 0.06 1.94 (0.96-3.90) 0.06 1.84 (0.81-4.14) 0.08

DP2 1.52 (0.85-2.72) 0.16 1.56 (0.84-2.90) 0.16 1.68 (0.88-3.21) 0.12 1.95 (0.98-3.89) 0.06

DP3 (ref) 1 1 1 1

*3-year* *prevalent sarcopenia*

n 183 179 179 175

DP1 1.66 (0.73-3.76) 0.23 2.50 (1.02-6.12) 0.045 2.44 (0.95-6.32) 0.07 2.07 (0.75-5.68) 0.16

DP2 2.45 (1.08-5.56) 0.03 2.46 (1.05-5.78) 0.04 2.71 (1.10-6.64) 0.03 3.62 (1.33-9.88) 0.01

DP3 (ref) 1 1 1

*3-year incident sarcopenia*

n 147 143 143 142

DP1 1.10 (0.35-3.38) 0.89 1.41 (0.41-4.90) 0.59 1.18 (0.31-4.54) 0.81 0.71 (0.16-3.19) 0.66

DP2 1.81 (0.60-5.50) 0.29 1.67 (0.53-5.23) 0.38 1.40 (0.41-4.83) 0.59 2.02 (0.79-7.81) 0.31

DP3 (ref) 1 1 1 1

DP1 ‘Low Red Meat’; DP2 ‘Traditional British’; DP3 ‘Low Butter’.

^a^Sarcopenia status was determined using the European Working Group on Sarcopenia in Older People (EWGSOP) definition as described previously [4]. ^b^Data from two waves (2006/07 to 2009/10) were used for incidence sarcopenia. n indicated the number of participants with complete data (sarcopenia status (yes/no), DP and risk factors).

OR, odds ratios; CI, confidence intervals; DPs, dietary patterns; ref, reference group; BW, body weight

Model 1 is unadjusted.

Model 2 is adjusted for socio-demographic factors (sex, social class, education).

Model 3 is additionally adjusted for health-related factors (cognitive status, depressive symptoms, total number of diseases, and total number of medication).

Model 4 is further adjusted for lifestyle factors (physical activity, smoking and food energy).
